# Cardiovascular disease in thymic cancer patients

**DOI:** 10.3389/fcvm.2024.1393631

**Published:** 2024-09-10

**Authors:** Abhishek Khemka, Suparna C. Clasen, Patrick J. Loehrer, Anna R. Roberts, Lilian Golzarri-Arroyo, Sunil S. Badve, Subha V. Raman, Siu L. Hui, Titus K. L. Schleyer

**Affiliations:** ^1^ Division of Cardiology, Department of Medicine, Indiana University School of Medicine, Indianapolis, IN, United States; ^2^ Department of Medicine, Simon Cancer Center, Indiana University School of Medicine, Indianapolis, IN, United States; ^3^ Regenstrief Data Services, Regenstrief Institute, Indianapolis, IN, United States; ^4^ School of Public Health, Indiana University Bloomington, Bloomington, IN, United States; ^5^ Department of Pathology, Emory School of Medicine, Atlanta, GA, United States; ^6^ Heart and Vascular Services, Ohio Health, Columbus, OH, United States; ^7^ Center for Biomedical Informatics, Regenstrief Institute, Indianapolis, IN, United States; ^8^ Division of Internal Medicine and Geriatrics, Department of Medicine, Indiana University School of Medicine, Indianapolis, IN, United States

**Keywords:** thymic cancer, cancer survivorship, cardiovascular disease, electronic health records, thoracic oncology, cardiovascular risk factors

## Abstract

**Introduction:**

Cancer patients may have increased risk for adverse cardiac events, but our understanding of cardiovascular risk in thymic cancer patients is not clear. We sought to characterize baseline cardiometabolic risk factors before thymic cancer diagnosis and the potential association between cancer treatment and subsequent cardiac events.

**Methods:**

This was a retrospective cohort study evaluating patients with thymic cancer from 2003 to 2020 compared to age- and sex-matched controls without cancer. Baseline cardiovascular risk factors, cancer characteristics, and incidence of cardiac events were collected from the health information exchange. Multivariable regression was used to examine the impact of cardiovascular risk factors and cancer therapies.

**Results:**

We compared 296 patients with pathology-confirmed thymic cancer to 2,960 noncancer controls. Prior to cancer diagnosis, thymic cancer patients (TCPs) had lower prevalence of hypertension, dyslipidemia, and diabetes mellitus and similar rates of obesity, tobacco use, and pre-existing cardiovascular disease (CVD) compared to controls. After diagnosis, high-risk TCPs (>2 cardiovascular risk factors or pre-existing CVD) had higher risk for cardiac events (HR 3.73, 95% CI 2.88–4.83, *p* < 0.001). In the first 3 years after diagnosis, TCPs had higher incidence of cardiac events (HR 1.38, 95% CI 1.01–1.87, *p* = 0.042). High-risk TCPs who received radiotherapy or chemotherapy had higher risk of cardiac events (HR 4.99, 95% CI 2.30–10.81, *p* < 0.001; HR 6.24, 95% CI 2.84–13.72, *p* < 0.001).

**Discussion/conclusion:**

Compared to noncancer controls, TCPs experienced more cardiac events when adjusted for risk factors. Patients with multiple cardiovascular risk factors receiving radiotherapy or chemotherapy had higher incidence of cardiac events.

## Introduction

Although survival from thymic cancer is relatively high, not much is known about cardiovascular (CV) outcomes. Thymic cancers are rare, with 1.5 cases occurring for every million people per year (1). Five-year survival for thymoma, thymic carcinoma, and thymic neuroendocrine tumors nears 90%, 55%, and 28%–75%, respectively (2–4). A third of patients have concomitant paraneoplastic syndromes such as myasthenia gravis, but there are limited data on adverse cardiovascular events in this subpopulation (5–7). Multimodal therapy for thymic tumors includes surgical resection potentially involving cardiac structures, cytotoxic chemotherapy (e.g., anthracyclines, platinum therapies), immunotherapies, and radiotherapy (with the heart possibly in the treatment field) (8). Side effects from cancer therapies can lead to significant cardiovascular morbidity and mortality (9) through direct cardiovascular toxicity—aggravating CV risk factors or exacerbating underlying cardiovascular disease (CVD) (10–14). To date, case reports of patients with thymic cancer have observed pericardial effusion, myocarditis, and constriction (15–21). Development of CV complications after therapy is poorly understood in thymic cancer patients. This risk may be increased in patients with underlying cardiometabolic risk factors and tobacco use (22).

The goal of this study was to assess whether patients with thymic cancer had an increased incidence of CVD compared to age- and sex-matched noncancer controls. Our comparison focused on the following questions: (1) Was the cancer's presence associated with higher CV risk factors before diagnosis and higher incidence of adverse cardiac events after treatment? (2) If there was a higher incidence of CVD, did the risk of cardiac events increase with increasing CV risk factors, and was the increased risk of cardiac events associated with clinical characteristics or cancer therapies? (3) Finally, did having a simultaneous paraneoplastic syndrome lead to an increased incidence of CVD?

## Methods

We conducted a retrospective analysis comparing cardiovascular outcomes in patients with a history of thymic cancer to noncancer patients from 2003 to 2020. Our referral center partnered with the Regenstrief Institute to evaluate data from the Indiana Network for Patient Care (INPC), a statewide health information exchange (23). This study received the proper ethical oversight and was approved by the Indiana University Institutional Review Board (# 2010183517). The reporting in this manuscript adheres with the STROBE guidelines (see Supplementary Material for checklist).

### Data sources

Medical and pharmacy data, notes, laboratory results, and cardiovascular imaging were obtained from the Indiana University (IU) Health Data Warehouse and INPC. The INPC, managed by the Indiana Health Information Exchange, is one of the largest health information exchanges in the country (24). Over 123 separate clinical entities contribute data representing 19 million patients, billions of clinical observations, and over 300 million clinical text reports. The INPC contains data for two-thirds of Indiana's population—a proportion that rises to 80%–100% in central Indiana where the major health systems are located (25, 26). INPC data were complemented with data from the IU Health Data Warehouse, which contains structured data from IU Health's Cerner electronic health records (EHR). As of late 2022, this database captures data on 5.6 million patients, representing 16 hospitals and numerous outpatient clinics.

### Data collection

**Figure 1:**
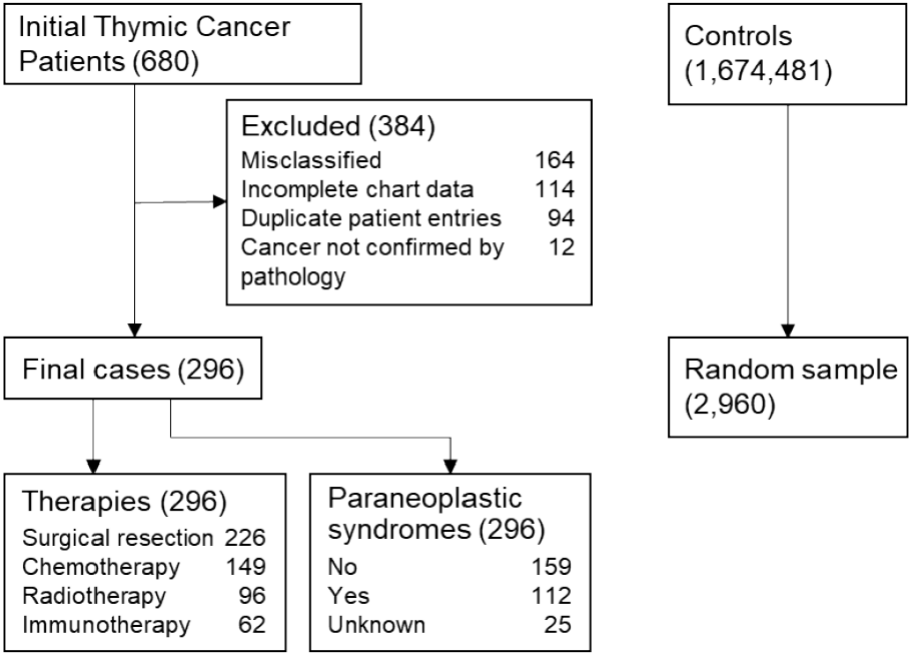
Consort diagram of thymic cancer patients and controls. There were initially 680 patient charts reviewed from which 296 patients were ultimately included for the final analysis. They were age-, sex- matched in a 1:10 fashion to 2,960 controls for prevalence of cardiac risk factors and cardiac outcomes. Cancer patients were analyzed for cardiac events based on their cancer therapies and presence or absence of a paraneoplastic syndrome.

Eligible patients over the age of 35 at the time of thymic cancer diagnosis were identified by ICD-9 (164.0, 212.6) and ICD-10 (C37, D15.0) codes for thymoma, thymic carcinoma, and thymic neuroendocrine tumor (Figure 1; see Supplementary Material for the complete ICD codes list). Patients with fewer than 2 ICD diagnoses of thymic tumor in the EHR were excluded, and data were collected for two years prior to the index date. We identified 1,212 patients with thymic tumors from January 1, 2003, to December 31, 2020. Selecting patients residing in Indiana or a neighboring state allowed for closer follow-up and ensured adequate data collection but reduced the number to 680. Cancer characteristics were extracted from EHR data, including WHO staging, Masaoka staging, size of tumor, cancer therapies, and presence of a paraneoplastic syndrome. The tumor size was based on a CT scan at the time of diagnosis or final pathologic evaluation. Cardiovascular risk factors evaluated included diabetes mellitus, hypertension, dyslipidemia, obesity classified as BMI >30, and a history of tobacco use. Patients were noted to have pre-existing coronary disease if they had a diagnosis of coronary atherosclerosis/calcification from a CT scan or coronary angiogram within 1 year of thymic cancer diagnosis. Cardiac events included heart failure (including preserved and reduced ejection fraction), myocardial infarction or ischemic heart disease, stroke or transient ischemic attack, and atrial fibrillation. Patient mortality was ascertained from hospital discharge summaries and the Indiana State Department of Health's death certificate files.

All 680 patient records were individually reviewed by the first author (AK). Records were ascertained for the accuracy of diagnoses and cancer characteristics based on pathology data, imaging, and oncology notes. The list was cross-referenced with a separate internal pathologic database of patients from 2010 to 2020 (*n* = 155, 52.36%) to confirm thymic neoplasm. To verify the accuracy of the chart review, a blinded reviewer (SCC) was given records of 20/680 patients, half of whom had tissue biopsy confirming diagnosis and half did not, and accurately verified 19/20 (95.0%) of the diagnoses. Unclear diagnoses (*n* = 22) were adjudicated by two authors (PJL, SB) based on pathologic data. Cardiovascular risk factors and outcomes were captured from various settings including inpatient and outpatient clinical encounters, and at different Indiana institutions through the INPC. Although there were cancer patients from neighboring states, they continued to receive subspecialty care at the Simon Cancer Center years after their cancer diagnosis, providing reliable estimates of long-term outcomes.

A control cohort consisted of patients meeting the following criteria: no prior cancer diagnoses and at least 2 years of clinical data in the system prior to an encounter occurring the same year as a thymic cancer patient (TCP) index date. The study index date of a cancer patient was defined as the date of the first documented diagnosis of thymic neoplasm. The index date of a control patient was the first visit date in the same calendar year as the index date of the matched patient. Controls were randomly selected and were age and sex-matched to TCPs at a 10:1 ratio. Data extraction was repeated for the control cohort.

### Statistical analysis

Demographic and clinical characteristics of both cohorts are presented using the mean, standard deviation, or count unless otherwise specified, and between-group comparisons used the unpaired *t*-test or chi-squared test, respectively. Incident events (cardiac or death) were observed from the index date and censored at the last recorded encounter at IU Health. These events could be predicted by the multiple CV risks at baseline, which were then grouped into three risk levels (low, medium, high) to streamline subsequent analyses (see Supplementary Material Table S5). Time-to-event comparisons between groups were graphed using Kaplan-Meier curves and analyzed using causal-specific Cox regression (results presented as hazard ratio [HR] with 95% confidence interval [CI]), adjusted for age, sex, race, and the 3 CV risk levels. The cumulative incidence plots (Figure 3) showed potential change-points of the proportional hazards at around three years and 7 years. With the data censored at 7 years due to insufficient number of later events, the model with time-dependent hazards (with change-point at three years) showed significantly better fit than the constant hazards model (*p* < 0.025).

## Results

### Cancer characteristics

**Figure 2:**
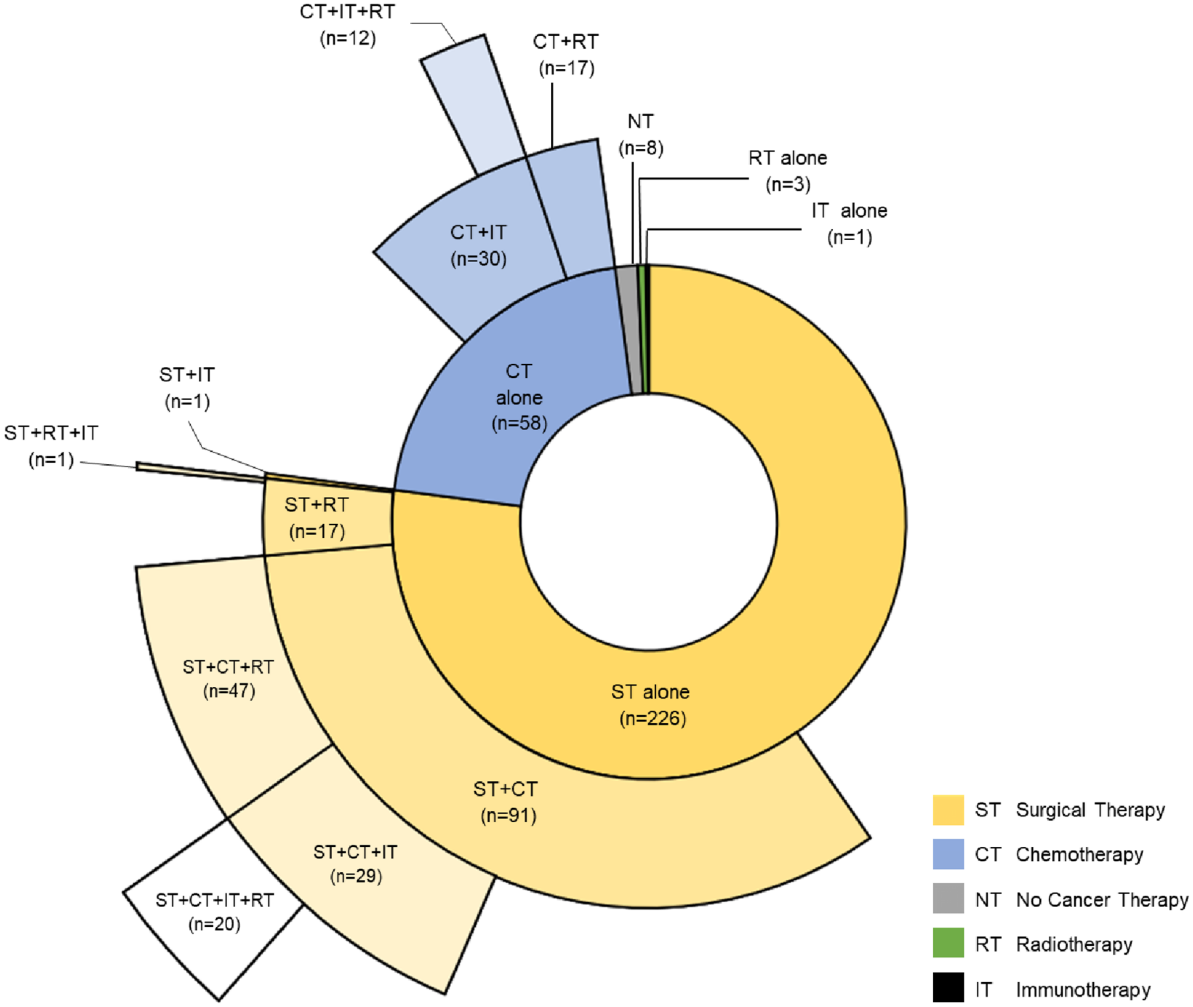
Distribution of cancer therapies for cohort of patients with cancer. Sunburst diagram depicting the type of cancer therapies and their combinations thymic cancer patients received.

Of the 1,212 patients identified with a thymic cancer diagnosis, 680 met the inclusion criteria. After excluding patients misclassified as having thymic cancer, duplicate entries from different health systems, patients with insufficient follow-up data, and patients without pathology-confirmed thymic cancer, the final cohorts consisted of 296 TCPs and 2,960 noncancer controls (Figure 1). Most TCPs had thymoma (74.0%), followed by thymic carcinoma (22.3%) and thymic neuroendocrine tumor (3.7%) (Table 1). Of 271 TCPs with Masaoka staging, 17.9%, 20.3%, 12.8%, and 40.5% were in stages 1–4, respectively. Paraneoplastic syndromes were present in 112 (37.8%) TCPs and 72 (24.3%) had myasthenia gravis. Treatments included surgical resection (76.4%), chemotherapy (50.3%) with 98 (33.1%) receiving anthracyclines, chest radiotherapy (32.4%), and immunotherapy (21.0%) (Figure 2).

**Table 1:** Cancer characteristics of cohort of patients with thymic cancer.

Cancer classification		n	%
Type of thymic cancer	Thymoma	219	73.99
	Thymic carcinoma	66	22.30
	Thymic Neuroendocrine tumor	11	3.72
Stage	I	53	17.91
	II	60	20.27
	III	38	12.84
	IV	120	40.54
	Unknown	25	8.45
Type of therapy	Presence	n	%
Surgical resection	Yes	226	76.35
	No	45	15.20
	Unknown	25	8.45
Chemotherapy	Yes	149	50.34
	No	122	41.22
	Unknown	25	8.45
Radiotherapy	Yes	96	32.43
	No	147	49.66
	Unknown	53	17.91
Immunotherapy	Yes	62	20.95
	No	140	47.30
	Unknown	94	31.76
Cancer attributes	Presence	n	%
Paraneoplastic syndrome	Yes	112	37.84
	No	159	53.72
	Unknown	25	8.45
Recurrent thymic neoplasm	Yes	83	28.04
	No	79	26.69
	Unknown	134	45.27
Previous history of another cancer	Yes	43	14.53
	No	75	25.34
	Unknown	178	60.14

Characteristics of cancer patients (296) including the subtype of thymic cancer, stage, type of cancer therapies received (mutually nonexclusive), and cancer attributes.

### Baseline characteristics of thymic cancer patients and controls

Age and sex distributions were similar for TCPs and controls (due to matching), and both groups were about 82% White (Table 2). Of the 296 patients, 198 (67%) were in Indiana and 98 (33%) were from neighboring states.

**Table 2:** Demographic data and cardiovascular risk factors of thymic cancer patients and patients without cancer (control group).

Demographic data and cardiovascular risk factors	TCPs (*n* = 296)		Control (*n* = 2,960)		*p*-value
Age					
Mean age (SD)	57.6	12.3	57.4	12.4	0.73
Median (min, max)	57	35,86	57	34,86	
Gender	n	%	n	%	>0.99
Female	149	50.34	1,490	50.34	
Male	147	49.66	1,470	49.66	
Race/ethnicity					0.005
White	244	82.43	2,423	81.86	
Black	26	8.78	385	13.01	
Other^a^	26	8.78	152	5.14	
Cardiovascular risk factors					
Diabetes mellitus	32	10.81	582	19.66	<0.001
Hypertension	94	31.76	1,449	48.95	<0.001
Dyslipidemia	76	25.68	1,085	36.66	<0.001
Obesity (BMI > 30)	33	11.15	419	14.16	0.18
Tobacco use	140	47.30	1,275	43.07	0.18
Preexisting cardiovascular disease	41	13.85	493	16.66	0.25
Number of CV Risk factors					<0.001
No previous CV event of CV risk factors	105	35.47	735	24.83	
1–2 CV risk factors but no prior CV event	109	36.82	1,219	41.18	
>2 CV risk factors and/or prior CV event	82	27.70	1,006	39.99	

TCPs, thymic cancer patients; CV, cardiovascular.

^a^ Other includes Asian/Pacific Islander, Hispanic, American Indian/Alaska Native, or not identified.

### Cardiovascular risk factors and diseases before diagnosis

Compared to controls, TCPs had similar rates of baseline obesity, tobacco use, and pre-existing CVD but significantly lower rates of hypertension, dyslipidemia, and diabetes mellitus (Table 2) and lower total number of CV risk factors per person. Preliminary analyses of TCPs showed these risk factors were predictive of subsequent cardiac events, and TCPs could be grouped into three levels of overall CV risk: 1) low-risk (no risk factors or pre-existing CVD); 2) medium risk (1–2 risk factors and no pre-existing CVD); and 3) high risk (>2 risk factors and/or pre-existing CVD).

### Cardiac events after cancer diagnosis

Over a mean follow-up period of 4.7 years (median = 4.4, range 0.5–16 years), the following events occurred: 31 TCPs and 346 controls developed heart failure (10.5% vs. 11.7%); 33 TCPs and 286 controls developed atrial fibrillation/flutter (11.2% vs. 9.7%); 14 TCPs and 195 controls had a stroke (4.7% vs. 6.6%); 15 TCPs and 259 controls had an ischemic event/myocardial infarction (5.1% vs. 8.8%); 63 TCPs and 686 controls had any cardiac event (21.3% vs. 23.2%); and 19 TCPs and 91 controls died (6.4% vs. 3.1%) (see Supplementary Material Table S5).

### Prediction of incident cardiac events in thymic cancer patients and controls

**Figure 3:**
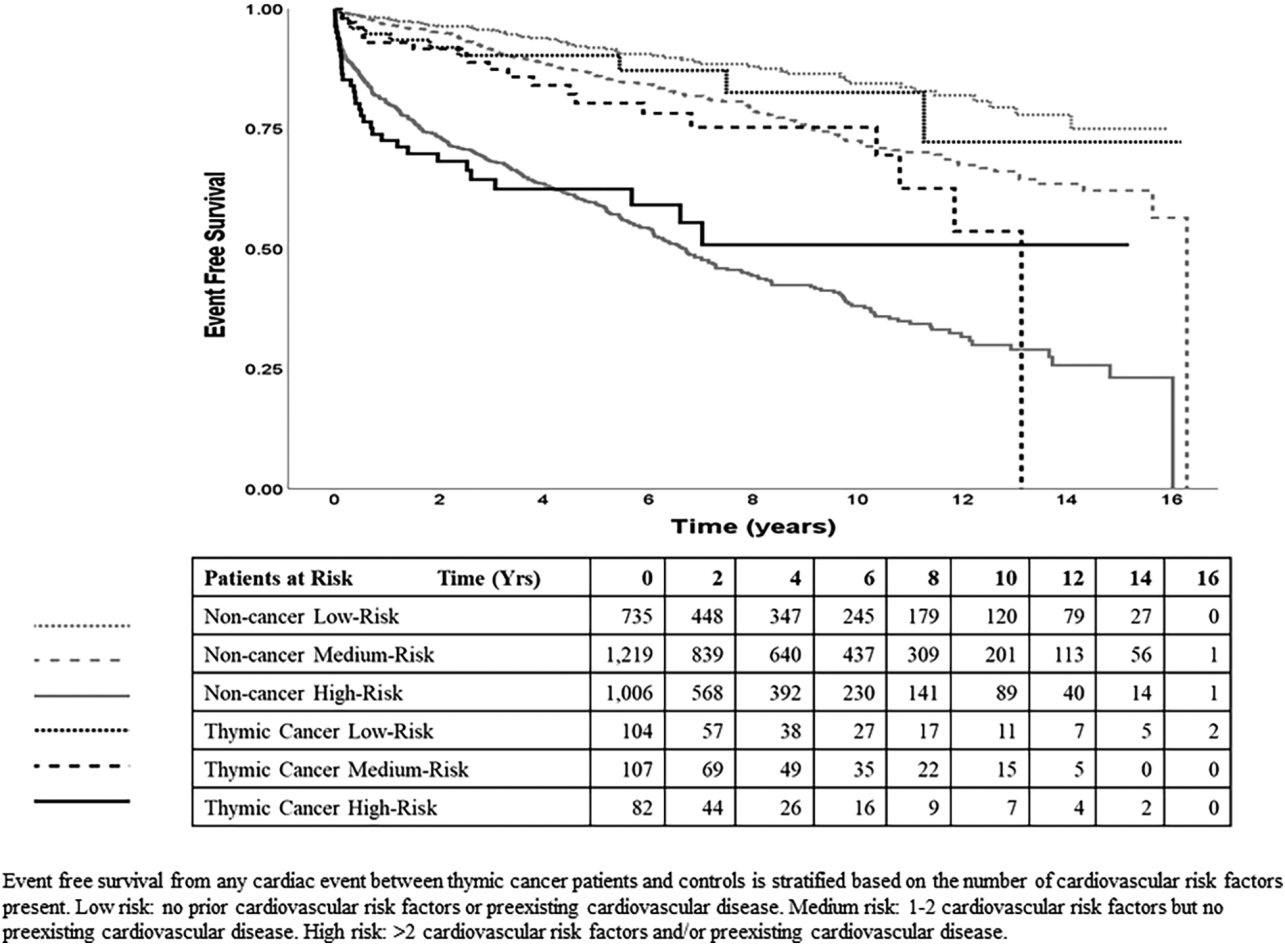
Event free survival of thymic cancer patients compared to controls.

The Kaplan-Meier curves (Figure 3) show that within TCPs and within noncancer controls, the incidence of CV events increases with the levels of baseline CV risk (low, medium, high) and within each risk level, the incidence of CV events was higher in TCPs than in controls. Results of the final time-dependent proportional hazards model showed that higher-incident cardiac events could be predicted by increasing age (HR = 1.05 per year) and male sex (HR = 1.37 relative to female) (Table 3). The 3 CV risk levels at baseline were also strongly predictive of the incidence of cardiac events, with HR of 1.45 (95% CI 1.11–1.90, *p* = 0.007) for medium risk and 3.73 (95% CI 2.88–4.83, *p* < 0.001) for high relative to low risk. The hazard ratios of TCPs relative to controls were 1.38 (95% CI 1.01–1.87, *p* = 0.042) in years 0–3 and 0.77 (95% CI 0.47–1.25, *p* = 0.29) in years 4–7.

**Table 3:** Cox regression comparing thymic cancer patients (TCPs) to controls with time-dependent hazard ratios of any cardiac event as the outcome.

Predictors	Hazard ratio (95% CI)	*p*-value
Pre-existing CV risk level compared to low risk)^a^		
Medium risk	1.45 (1.11–1.90)	0.007
High risk	3.73 (2.88–4.83)	<0.001
Demographics		
Age (years at index date)	1.05 (2.88–4.83)	<0.001
Sex (male vs. female)	1.37 (1.18–1.58)	<0.001
Race (compared to white)		
Black	1.13 (0.91–1.39)	0.27
Other	0.81 (0.53–1.25)	0.34
TCPs vs. controls (0–3 years)	1.38 (1.01–1.87)	0.042
TCPs vs. controls (>3 years)	0.77 (0.47–1.25)	0.29

TCPs, thymic cancer patients; CV, cardiovascular.

Higher-incident cardiac events could be predicted by increasing age (HR = 1.05 per year) and male sex (HR = 1.37 relative to female). The 3 CV risk levels at baseline were also strongly predictive of the incidence of cardiac events, with HR of 1.45 for medium risk and 3.73 for high relative to low risk. The hazard ratios of TCPs relative to controls were 1.38 in years 0–3 and 0.77 in years 4–7. “Time-dependent” refers to likelihood ratio test with chi-square (1 df) = 4.3, *p* < 0.05, showing significantly better fit than constant hazards model.

^a^ Low risk: no prior cardiovascular risk factors or preexisting cardiovascular disease. Medium risk: 1–2 cardiovascular risk factors but no preexisting cardiovascular disease. High risk: > 2 cardiovascular risk factors and/or preexisting cardiovascular disease.

### CVD outcomes as related to treatments in thymic cancer patients

To assess the validity of our data source and approach, we evaluated the known association of heart failure with anthracycline therapy in our dataset. In our cancer cohort (12 heart failures among 98 anthracycline-treated patients and 19 heart failures among 198 non-treated patients), anthracycline-treated patients had significantly higher overall heart failure events (HR = 2.77, 95% CI 1.26–6.06, *p* = 0.011) after controlling for demographics and baseline CV risk. Among classes of cancer therapies, immunotherapy showed no significant association with cardiac events among the cancer patients with time-dependent analysis. The effects of radiotherapy and chemotherapy were time-dependent. There was a significantly higher incidence of any cardiac event with radiotherapy and chemotherapy treatments compared to untreated cancer patients, but this difference did not manifest until after the first three years (Table 4). The data were too limited to investigate the effects of treatment combinations.

**Table 4:** Cox regression with time-dependent hazard ratios for any cardiac event, halted at 7 years, comparing cancer patients that received the specific cancer therapy to cancer patients who did not receive the therapy.

Predictors	Radiotherapy		Chemotherapy		Immunotherapy	
	Hazard ratio (95% CI)	*p*-value	Hazard ratio (95% CI)	*p*-value	Hazard ratio (95% CI)	*p*- value
Pre-existing CV risk level (compared to low risk)^a^						
Medium risk	1.68 (0.74–3.77)	0.21	1.77 (0.78–3.99)	0.17	1.77 (0.85–3.68)	0.13
High risk	4.99 (2.30–10.81)	<0.001	6.24 (2.84–13.72)	<0.001	3.68 (1.80–7.49)	<0.001
Demographics						
Age (years at index date)	1.03 (1.00–1.05)	0.03	1.03 (1.00–1.05)	0.018	1.03 (1.01–1.05)	0.010
Sex (male vs. female)	0.81 (0.48–1.39)	0.45	0.79 (0.46–1.34)	0.38	0.94 (0.57–1.57)	0.82
Race (compared to white)						
Black	0.60 (0.21–1.70)	0.34	0.54 (0.19–1.52)	0.24	0.53 (0.19–1.50)	0.23
Other	0.20 (0.03–1.48)	0.12	0.18 (0.03–1.33)	0.09	0.19 (0.03–1.35)	0.010
Treated vs. untreated cancer patients (0–3 years)	1.50 (0.79–2.83)	0.21	1.94 (1.04–3.62)	0.038	0.77 (0.32–1.84)	0.55
Treated vs. untreated cancer patients (>3 years)	7.03 (1.80–27.42)	0.005	7.43 (1.89–29.25)	0.004	1.84 (0.52–6.52)	0.35

TCPs, thymic cancer patients; CV, cardiovascular.

There was a time-dependent difference between TCPs treated and TCPs not treated with specific cancer therapies. There was significantly higher incidence of any cardiac event with radiotherapy and chemotherapy treatments compared to controls, but this difference did not consistently manifest until after the first 3 years “Time-dependent” refers to likelihood ratio test with chi-square (1 df) = 6.25, *p* = 0.0124 for radiotherapy and chi-square (1 df) = 7.03, *p* = 0.0080 for chemotherapy, showing significantly better fit than constant hazards model for each therapy.

^a^ Low risk: no prior cardiovascular risk factors or preexisting cardiovascular disease. Medium risk: 1–2 cardiovascular risk factors but no preexisting cardiovascular disease. High risk: > 2 cardiovascular risk factors and/or preexisting cardiovascular disease.

Secondary analyses comparing TCPs with and without a paraneoplastic process after cancer diagnosis showed no significant difference in adverse CVD or death (HR 0.71, 95% CI 0.41–1.23, *p* = 0.22; HR 1.24, 95% CI 0.5–3.09, *p* = 0.64).

## Discussion

The results of this single-center, retrospective cohort study indicate that patients with thymic neoplasms may have had a lower prevalence of certain cardiovascular risk factors compared to non-cancer controls before their cancer diagnosis. This unexpected finding, along with the increased risk of CVD within the first three years after thymic cancer diagnosis, suggests there may be underlying mechanisms in thymic cancer that accelerate cardiac disease, warranting further investigation. This potential discrepancy could be attributed to incomplete data collection before the diagnosis of thymic cancer, possibly influenced by referral bias. We hope to compare these findings of lower baseline cardiovascular risk factors in patients with thymic cancer to a larger multicenter database to assess their validity. It is important to note that TCPs had a similar incidence of cardiac events compared to controls during follow-up after their cancer therapies. When adjusted for age, sex, race, and number of CV risk factors, the risk for any cardiac event was significantly higher in the cancer cohort. After grouping TCPs into three risk strata based on CV risk factors and pre-existing disease, we found an additive risk for cancer patients between the number of risk factors and incident cardiac events. In the three years after diagnosis, TCPs had a higher incidence of cardiac events than controls, regardless of baseline risk factors or cancer therapy. After three years, TCPs who received radiotherapy or chemotherapy had a higher incidence of cardiac events compared to untreated TCPs.

We did not match cohorts based on CV risk factors due to the exploratory nature of the study. By not matching, in this limited dataset, we observed that TCPs had a lower total number of baseline risk factors than matched controls in this cohort. In high-risk patients with >2 underlying risk factors or pre-existing CVD, the risk of heart failure with anthracyclines was significantly higher. Similarly, in patients receiving radiotherapy or any chemotherapy, the risk of any cardiac event was significantly higher in high-risk cancer patients. The finding of a higher risk of cardiac events in TCPs in the first three years compared to controls is interesting. TCPs may have had pre-existing heart disease that went unnoticed before their cancer diagnosis, or they may have undergone surgery impacting cardiac structures, resulting in subsequent injury or arrhythmias. A less probable scenario to consider is that some TCPs with a paraneoplastic syndrome might have experienced myocarditis, although a prior review found this to be uncommon (27). In our study, there was one TCP with myocarditis. This occurrence may be attributed to the involvement of inflammatory cytokines implicated in cardiac events in cancer patients which may have contributed (28). While there is an apparent indication of increased CVD in the initial three years for TCPs, this might be attributed to intensified monitoring because cardiac events resulting from radiotherapy and chemotherapy typically require time to manifest. As patients undergo cancer therapy, they may experience multiple CV stressors in combination with lifestyle factors and metabolic dyscrasias, potentially contributing to the development of CVD and premature mortality (29).

Our study adds important information to the current paradigms regarding cardiac events in patients with thymic neoplasms. The increased prevalence of cardiac events is observed not only in thymic cancer patients but also in those with other types of cancer as demonstrated by several studies. Armenian et al. (14) found that patients with multiple myeloma, non-Hodgkin lymphoma, kidney cancer, and breast cancer have significantly higher incidence of CVD when compared to noncancer controls. Other studies have similarly reported an elevated risk of CVD in patients with cancers sharing characteristics with thymic cancer, namely a thoracic location and similar therapeutic regimens with chemotherapy (e.g., anthracyclines) and radiation with the heart in the treatment field. These malignancies include lung cancer (12), breast cancer (13), and mediastinal lymphomas (30, 31). CVD and lung cancer share common risk factors, including tobacco use and inflammation, and pre-existing CVD before cancer therapy portends a worse prognosis (12). Complications from cancer therapies for thoracic tumors include hypertension, coronary disease, myocarditis, and arrhythmias. Certain chemotherapies such as cyclophosphamide may not immediately lead to cardiotoxicity but may activate endothelial dysfunction and trigger long term cardiac complications (32, 33). Furthermore, for these other thoracic cancers, the risk increases based on several baseline risk factors, total heart dose of radiotherapy, and additive effects from multiple therapies such as anthracyclines and other cardiotoxic agents (34–36). However, ours is the first, relatively large, comprehensive study evaluating CV risk factors and CVD in patients with pathology-confirmed thymic cancers. In a study by Okereke et al., 11% of patients with recent diagnoses of thymomas had a known history of cardiac disease—similar to our own (13.9%), although in their study, stroke was not included (37). In a smaller study by Liao et al., postoperative radiotherapy improved survival from thymomas but increased the risk of cardiac events in long-term survivors (38).

The mechanism for why TCPs had an increased incidence of cardiac events is not entirely clear. We speculate that potential cardiotoxic treatments lead to or exacerbate underlying CVD, especially in the presence of multiple CV risk factors. Almost half of the patients had a history of tobacco use, and a substantial number had hypertension, diabetes, and dyslipidemia. Chemotherapy and radiotherapy may lead to changes in blood pressure, metabolic factors, and increased inflammation (39–42). Patients receiving left-sided radiotherapy are at increased risk for developing coronary disease, valvulopathies, and cardiac death (43). Immunotherapy-related adverse events have been observed, with myocarditis being the most notable although other reported toxicities include cardiomyopathy, conduction defects, and pericarditis. Long-term complications and the value of screening and surveillance approaches for immunotherapy patients have not been delineated (44). There are several clinical implications from our data in the care of thymic patients: TCPs may benefit from more aggressive control of baseline CV risk factors prior to and during cancer treatments. There may be value in providing cardiac evaluation prior to treatment and ongoing cardiac monitoring, especially in high-risk patients receiving chemotherapy or radiotherapy. In high-risk patients, oncologists may avoid adjuvant therapy if there is minimal impact on cancer survival. These changes may lead to improved morbidity and mortality in TCPs.

Our study's strengths include having the largest cohort of pathology-confirmed TCPs for evaluation of CVD to date and using a statewide health information exchange to obtain comprehensive data. Our study is the first to evaluate baseline CV risk factors and appraise subsequent cardiac events. Previous studies have had smaller patient populations with shorter follow-ups and did not account for cardiometabolic risk factors. Given the paucity of comprehensive data in the field and that ours is a single-center, observational study, we advocate for a larger multicenter to validate our findings, including the timing of cardiac events. A prospective study evaluating the benefit of cardiac evaluation and preemptive interventions for cardiovascular risk factors prior to therapy may help determine benefits for CVD and survival.

### Limitations

Our study had several limitations. It featured a single quaternary referral center's experience and included many patients with advanced-stage tumors. This may have skewed the number of cardiac events. Less complete historical data from referral patients may have biased our results. There may have been bias based on the geographical distribution of referrals as rural areas tend to provide less data to the INPC. We may have limited data prior to the index date and missed pre-existing conditions. Selection bias may have resulted from evaluating only patients over 35 and limiting the cohort to patients from Indiana or a neighboring state. By limiting the cohort geographically, we aimed to obtain comparatively more complete data with longitudinal follow-up due to the high penetration of our health information exchange. To ensure accuracy, we minimized potential recall and misclassification biases using both ICD coding and physician review of EHR data. We strove to have accurate diagnoses by only including patients with pathologic diagnoses of thymic neoplasm and confirmed with a subset of patients with a second pathology database. This led to smaller numbers of patients in the study, decreasing the power for multivariable analyses. Patients with thymic cancers in our institution have not traditionally had extensive cardiac workups prior to initiating treatment or undergoing surgical resection, which may have led to underestimation of baseline CVD. We could not separate exposure from treatments, such as concurrent chemotherapy and immunotherapy, to analyze cardiac events which likely led to confounding. We had similar distribution of thymic cancer subtypes and similar incidence of cardiac events compared to the published literature. Our subanalysis for heart failure in TCPs who received anthracyclines also reinforces validity. Since this was an exploratory study, we did not perform propensity score matching. We cannot make causal inferences but were able to highlight associations between baseline CV risk factors and higher incidence of adverse CVD in TCPs.

## Conclusion

In our study, TCPs exhibited cardiac event rates comparable to controls. Stratification by baseline cardiovascular risk factors revealed an additive risk for cardiac events in TCPs. High-risk patients undergoing radiotherapy or chemotherapy experienced worse cardiac outcomes, underscoring the necessity for strategies to mitigate cardiovascular risk. Further studies are warranted to better understand specific associations contributing to increased cardiovascular events.

## Data Availability

The raw deidentified data supporting the conclusions of this article will be made available by the authors, without undue reservation.
