# Supplementary material for: Cardiovascular disease in thymic cancer patients
Source: Front Cardiovasc Med. 2024 Sep 10;11:1393631. doi: 10.3389/fcvm.2024.1393631 (PMC11427757; doi:10.3389/fcvm.2024.1393631)
Supplement: Supplementary file 1 [file Datasheet1.docx]

Supplementary Material

# Supplementary Figures and Tables

Table 5. Cancer patients’ risk of cardiac events (online only)

|  | **Cardiac Events in Thymic Cancer Patients** | **Cardiac Events in Control Patients** | **HR** | **95% Confidence Interval** | **p-value** |
| --- | --- | --- | --- | --- | --- |
| **Unadjusted Analysis of CVD** | | | | | |
| Heart Failure | 31 | 346 | 0.94 | 0.65, 1.36 | 0.75 |
| Atrial Fibrillation | 33 | 286 | 1.30 | 0.91, 1.87 | 0.15 |
| Stroke | 14 | 195 | 0.76 | 0.44, 1.3 | 0.31 |
| Heart Attack | 15 | 259 | 0.60 | 0.36, 1.01 | 0.055 |
| Any Cardiac Event | 63 | 686 | 0.99 | 0.76, 1.28 | 0.94 |
| Death | 19 | 91 | 2.35 | 1.43, 3.86 | <0.001 |
| **Risk for CVD based on the number of baseline CV risk factors, compared to low risk*** | | | | | |
| Medium risk | | | 1.45 | 1.11, 1.90 | 0.007 |
| High risk | | | 3.73 | 2.88, 4.83 | <0.001 |

In order to reduce the number of different CV risk predictors as covariates for subsequent models, we performed preliminary analyses that predicted incident CV events in all subjects using the number of pre-existing risks (0, 1, 2, >2), the presence of pre-existing CVD, and other covariates (demographics and TCP vs. controls). Based on the log odds of the risk predictors, we formed subgroups of patients with similar baseline risks and the final risk strata chosen were:

*Level 1: Low risk - 0 risk factors and no pre-existing CVD, Level 2: Medium risk - 1-2 risk factors and no pre-existing CVD, Level 3: High risk - >2 risk factors and/or presence of pre-existing CVD. CVD = cardiovascular disease, CV = cardiovascular

*ICD codes (online only):*

| **Condition** | **ICD-9** | **ICD-10** | | |
| --- | --- | --- | --- | --- |
| Diabetes (type II) | 250.0, 250.2 | E11* | | |
| HTN | 401*, 402*, 403*,  404* | I10*, I11*, I12*, I13* | | |
| Dyslipidemia | 272.4 | E78.5 | | |
| Obesity | 278* | E66* | | |
| Myasthenia gravis | 358.0* | G70.0* | | |
| Neuropathy | 337.0*, 337.1 | G90.0*, G99.0 | | |
| Myocarditis | 422*, 429.0,  074.23 | I40*, B33.22, I51.4 | | |
| Red call aplasia | 284.01, 284.81 | D60* | | |
|  |  |  | | |
|  |  |  | | |
| **Cardiac history (CVM definitions)** | |  | | |
|  |  |  | | |
| **Atrial Fibrillation** |  |  | | |
| **Condition** | **ICD-9** |  | |  |
| Atrial Fibrillation and Flutter | 427.3 |  | |  |
| Atrial Fibrillation | 427.31 |  | |  |
| Atrial Flutter | 427.32 |  |  |  |
| **Condition** | **ICD-10** |  |  |  |
| Atrial Fibrillation and Flutter | I48.* |  |  |  |
|  |  |  | | |
| **Congestive Heart Failure (CHF)** | |  | | |
| **Condition** | **ICD-9** |  | | |
| Rheumatic Heart Failure (congestive) | 398.91 |  | | |
| Malignant Hypertensive Heart Disease with Heart Failure | 402.01 |  | | |
| Benign Hypertensive Heart Disease with Heart Failure | 402.11 |  | | |
| Unspecified Hypertensive Heart Disease with Heart Failure and Stage I through IV CKD | 402.91 |  | | |
| Hypertensive Heart and CKD, Malignant, with Heart Failure and Stage I through IV CKD | 404.01 |  | | |
| Hypertensive Heart & CKD, malignant, with Heart Failure and Stage V or end stage renal disease | 404.03 |  | | |
| Hypertensive Heart and CKD, Benign, with Heart Failure and Stage I through IV CKD | 404.11 |  | | |
| Hypertensive Heart & CKD, benign, with Heart Failure and Stage V or end stage renal disease | 404.13 |  | | |
| Hypertensive Heart and CKD, unspecified, with Heart Failure  and Stage I through IV CKD | 404.91 |  | | |
| Hypertensive Heart & CKD, unspecified, with Heart Failure and Stage V or end stage renal disease | 404.93 |  | | |
| Heart Failure | 428.* |  | | |
| **Condition** | **ICD-10** |  | | |
| Rheumatic Heart Failure | I09.81 |  | | |
| Hypertensive Heart Disease with Heart Failure | I11.0 |  | | |
| Hypertensive Heart and CKD with Heart Failure and Stage 1 through IV CKD | I13.0 |  | | |
| Hypertensive Heart & CKD with Heart Failure and Stage V or end stage renal disease | I13.2 |  | | |
| Heart Failure | I50.* |  | | |
|  |  |  | | |
| **Coronary Artery Disease (CAD)** | |  | | |
| **Condition** | **ICD-9** |  | | |
| Acute myocardial infarction | 410.* |  | | |
| Other acute and subacute forms of ischemic heart disease | 411.* |  | | |
| Old myocardial infarction | 412.* |  | | |
| Other forms of chronic ischemic heart disease | 414.* |  | | |
| **Condition** | **ICD-10** |  | | |
| ST elevation (STEMI) and non-ST elevation (NSTEMI)  myocardial infarction | I21.* |  | | |
| Subsequent ST elevation (STEMI) and non-ST elevation  (NSTEMI) myocardial infarction | I22.* |  | | |
| Certain current complications following ST elevation (STEMI) and non-ST elevation (NSTEMI) myocardial infarction (within the 28-day period) | I23.* |  | | |
| Other acute ischemic heart diseases | I24.* |  | | |
| Chronic ischemic heart disease | I25.* |  | | |
|  |  |  | | |
| **Myocardial Infarction** | |  | | |
| **Condition** | **ICD-9** |  | | |
| Acute myocardial infarction | 410.* |  | | |
| Old myocardial infarction | 412.* |  | | |
| **Condition** | **ICD-10** |  | | |
| ST elevation (STEMI) and non-ST elevation (NSTEMI)  myocardial infarction | I21.* |  | | |
| Old myocardial infarction | I25.2 |  | | |
|  |  |  | | |
| **Stroke** |  |  | | |
| **Condition** | **ICD-9** |  | | |
| Cerebral thrombosis with cerebral infarction | 434.01 |  | | |
| Cerebral embolism with cerebral infarction | 434.11 |  | | |
| Cerebral artery occlusion, unspecified with cerebral infarction | 434.91 |  | | |
| Transient cerebral ischemia | 435.* |  | | |
| **Condition** | **ICD-10** |  | | |
| Cerebral Infarction | I63.* |  | | |

STROBE Statement—Checklist of items that should be included in reports of ***cohort studies***

|  | | Item No | Recommendation | Page No |
| --- | --- | --- | --- | --- |
| **Title and abstract** | | 1 | (*a*) Indicate the study’s design with a commonly used term in the title or the abstract | 1-2 |
|  |  |  | (*b*) Provide in the abstract an informative and balanced summary of what was done and what was found |  |
| Introduction | | | | |
| Background/rationale | | 2 | Explain the scientific background and rationale for the investigation being reported | 3 |
| Objectives | | 3 | State specific objectives, including any prespecified hypotheses | 3 |
| Methods | | | | |
| Study design | | 4 | Present key elements of study design early in the paper | 4-7 |
| Setting | | 5 | Describe the setting, locations, and relevant dates, including periods of recruitment, exposure, follow-up, and data collection | 4-7 |
| Participants | | 6 | (*a*) Give the eligibility criteria, and the sources and methods of selection of participants. Describe methods of follow-up | 4-7 |
|  |  |  | (*b*) For matched studies, give matching criteria and number of exposed and unexposed |  |
| Variables | | 7 | Clearly define all outcomes, exposures, predictors, potential confounders, and effect modifiers. Give diagnostic criteria, if applicable | 4-7 |
| Data sources/ measurement | | 8* | For each variable of interest, give sources of data and details of methods of assessment (measurement). Describe comparability of assessment methods if there is more than one group | 4-7 |
| Bias | | 9 | Describe any efforts to address potential sources of bias | 13 |
| Study size | | 10 | Explain how the study size was arrived at | 7 |
| Quantitative variables | | 11 | Explain how quantitative variables were handled in the analyses. If applicable, describe which groupings were chosen and why | 5-7 |
| Statistical methods | | 12 | (*a*) Describe all statistical methods, including those used to control for confounding |  |
|  |  |  | (*b*) Describe any methods used to examine subgroups and interactions | 6-7 |
|  |  |  | (*c*) Explain how missing data were addressed |  |
|  |  |  | (*d*) If applicable, explain how loss to follow-up was addressed |  |
|  |  |  | (*e*) Describe any sensitivity analyses |  |
| Results | | | |  |
| Participants | | 13* | (a) Report numbers of individuals at each stage of study—eg numbers potentially eligible, examined for eligibility, confirmed eligible, included in the study, completing follow-up, and analysed | 7-8 |
|  |  |  | (b) Give reasons for non-participation at each stage |  |
|  |  |  | (c) Consider use of a flow diagram |  |
| Descriptive data | | 14* | (a) Give characteristics of study participants (eg demographic, clinical, social) and information on exposures and potential confounders |  |
|  |  |  | (b) Indicate number of participants with missing data for each variable of interest | 7-9 |
|  |  |  | (c) Summarise follow-up time (eg, average and total amount) |  |
| Outcome data | | 15* | Report numbers of outcome events or summary measures over time | 9 |
| Main results | 16 | (*a*) Give unadjusted estimates and, if applicable, confounder-adjusted estimates and their precision (eg, 95% confidence interval). Make clear which confounders were adjusted for and why they were included | |  |
|  |  | (*b*) Report category boundaries when continuous variables were categorized | | 7-9 |
|  |  | (*c*) If relevant, consider translating estimates of relative risk into absolute risk for a meaningful time period | |  |
| Other analyses | 17 | Report other analyses done—eg analyses of subgroups and interactions, and sensitivity analyses | | 9 |
| Discussion | | | | |
| Key results | 18 | Summarise key results with reference to study objectives | | 9-10 |
| Limitations | 19 | Discuss limitations of the study, taking into account sources of potential bias or imprecision. Discuss both direction and magnitude of any potential bias | | 13 |
| Interpretation | 20 | Give a cautious overall interpretation of results considering objectives, limitations, multiplicity of analyses, results from similar studies, and other relevant evidence | | 11-12 |
| Generalisability | 21 | Discuss the generalisability (external validity) of the study results | | 12 |
| Other information | | | | |
| Funding | 22 | Give the source of funding and the role of the funders for the present study and, if applicable, for the original study on which the present article is based | | 20 |

*Give information separately for exposed and unexposed groups.

**Note:** An Explanation and Elaboration article discusses each checklist item and gives methodological background and published examples of transparent reporting. The STROBE checklist is best used in conjunction with this article (freely available on the Web sites of PLoS Medicine at http://www.plosmedicine.org/, Annals of Internal Medicine at http://www.annals.org/, and Epidemiology at http://www.epidem.com/). Information on the STROBE Initiative is available at http://www.strobe-statement.org.
